# Sepsis Management, Controversies, and Advancement in Nanotechnology: A Systematic Review

**DOI:** 10.7759/cureus.22112

**Published:** 2022-02-11

**Authors:** Rabia Choudhary

**Affiliations:** 1 Medicine, California Institute of Behavioral Neurosciences & Psychology, Fairfield, USA

**Keywords:** sepsis, nano particles, nano materials, nano-biotechnology, nano technology, sepsis treatment, antibiotic administration in management of severe sepsis

## Abstract

Sepsis is a potentially dangerous infection that requires prompt identification and treatment. Emergency medicine physicians must grasp the clinical signs and laboratory results of direct and indirect organ failure, the source of infection management, and the criteria for treating sepsis and septic shock. The pathogenesis of sepsis is connected to inflammation and an excess of reactive oxygen and nitrogen species, which activate the pathogen-associated molecular pattern (PAMP)-pattern recognition receptor (PRR) and damage-associated molecular pattern (DAMP)-PRR signaling pathways. The development of rapid, sensitive, and precise techniques for sepsis diagnosis might be aided by nanotechnology, a part of nanomedicine. Nanoparticles (NPs) such as magnetic NPs, gold NPs, fluorescent (silica and quantum dots), and lipid-based NPs have all been discussed to contribute to the detection of sepsis-related microbial infections. Because of the intrinsic and unique features of these nano-sized systems, researchers are evaluating nanotechnology-based alternatives for sepsis control. Recent advances in nanotechnology-based technologies for sepsis detection and management are discussed in this study.

Databases (PubMed, Medline, PMC, Google Scholar) were used to source various studies that were carried out on sepsis in terms of assessment, types, diagnosis, and treatment controversies, with more attention being given with a focus on the most recent data, principles, and management guidelines. Priority was also given to studies published within the last 11 years, using keywords such as "sepsis guidelines," "sepsis clinical," "septic risk factors," "sepsis and nano technology," "nano particles," "sepsis controversies," and "nano diagnostic" in the search. After a filtration process, the eight most relevant studies were selected to be included in this review. The filtration process included the use of both inclusion and exclusion criteria. The excluded studies were pediatric populations, obstetrical populations, and nanotechnology advancements dealing with other fields not relating to sepsis. The selected studies were also undertaken through a quality appraisal process using corresponding assessment tools. The selected articles were all highly informative about sepsis and the processes of diagnosis and treatment that are currently in use as well as those that are still being developed or implemented. Furthermore, we look at how nanomedicine in the application of nanomaterials can be employed to efficiently manage sepsis.

## Introduction and background

Septic shock is a major healthcare issue in many countries, with a high mortality rate. The mortality rate associated with septic shock has been estimated to be between 28% and 50 % in developed countries and higher (45 % to 74.6 %) in low-income countries. The primary cause of this has been identified as poor clinical diagnosis and management protocol for sepsis and its consequences, such as septic shock and multi-organ dysfunction, which have been pronounced during the surgical and anesthesia period [1].

Sepsis is a life-threatening condition that arises when the immune system's response to infection is altered. It is usually brought on by a systemic illness. Immune abnormalities generated by pathogenic microorganisms or tissue damage cause some aspects of the illness, culminating in organ failure and death. In its simplistic terms, the current definition recognizes the urgency of the term "sepsis," which is caused by an invading organism that results in an abnormal body reaction. Multi-organ failure is a combination of cardiovascular, cellular, coagulation, and endothelial dysfunction, and is sometimes referred to as "the four horsemen of the septic apocalypse" [1-2].

The World Health Assembly (WHA), the decision-making body of the World Health Organization (WHO), now recognizes sepsis as a serious danger to patient care and global health and hence has strengthened its approach to sepsis prevention, diagnosis, and management. It is accountable for approximately 20% of all deaths globally, and every year, nearly 1.7 million sepsis cases and 270,000 sepsis-related deaths are reported in the United States. Sepsis is a major cause of death and sickness in children under the age of five years, and it continues to be a prominent cause of intensive care unit admissions in developing countries [2].

Furthermore, research from the current coronavirus disease 2019 (COVID-19) pandemic has revealed a link between severe acute respiratory syndrome coronavirus 2 and sepsis, which emerges as a systemic inflammatory response and potentially organ damage due to viral invasion. As a result, sepsis remains a severe worldwide health problem with potentially fatal implications, requiring immediate attention, particularly early detection and effective therapeutic care [2].

Early detection of sepsis and prompt treatment intervention is critical for improving clinical outcomes and lowering mortality. Serum analysis and molecular methods have traditionally been used to diagnose sepsis. The diagnosis of sepsis is made more difficult by vague signs and symptoms, and therefore there is no gold-standard test that can confirm the diagnosis [3]. A blood culture test is the most frequent way to identify infectious bacteria in the bloodstream. Aside from that, various molecular approaches, such as polymerase chain reaction (PCR), and microarrays techniques are used to detect infection-causing microorganisms, each with varying sensitivity and specificity. Despite the critical need for sepsis monitoring using biomarkers in clinical diagnosis, there are currently no available sepsis-specific biomarkers. Over 170 biomarkers for sepsis detection have been identified, and only a few are useful in clinical diagnosis, and each has its own benefits and drawbacks [4-5].

The three major elements of sepsis care are (1) hemodynamic stabilization, (2) infection control, and (3) regulation of septic reactions. Generalized organ support therapies include oxygen therapy, mechanical ventilation, hemodynamic support, corticosteroids, and renal replacement therapy. Severity-based sepsis treatment necessitates multimodal therapeutic approaches. Multiple organ failure necessitates intrusive therapy, although a mild type with single organ system malfunction can be handled with minimal care. Although broad-spectrum antibiotics are essential in the treatment of sepsis, a drawback of antibiotic therapy in sepsis is pathogen resistance, which has a negative impact on sepsis outcomes and doubles fatality rates [2,6-9].

Nanotechnology is considerable potential for solving the aforementioned obstacles in sepsis management. In sepsis studies, nanoparticle (NP) based medication delivery of antibiotics has demonstrated encouraging outcomes in fighting drug resistance. By targeting pathogens or particular microenvironments, surface functionalization with antimicrobial peptides improves effectiveness. Various nano-formulation technologies that have shown the capacity to administer antibiotics and anti-inflammatory drugs at the same time have been discussed. Nanotechnology-based solutions are now being tested for the detection of infections and organ malfunctions, offering solutions in point-of-care settings for the diagnosis of immunological dysregulation [2,10].

The goal of this article is to provide current information and debates on sepsis care, while focusing on the most recent data, principles, and techniques. These are the evidence-based guidelines for sepsis and septic shock therapy. Fluids, steroids, early vasopressors, immunotherapy, and the development of nanotechnology are just a few of the current sepsis care debates that will be thoroughly discussed.

## Review

Methodology

This review adheres to the Preferred Reporting Items for Systematic Reviews and Meta-Analyses standards (PRISMA) [11]. Only peer-reviewed articles were included, and data was gathered from PubMed, Medline, PMC, and Google Scholar. Relevant publications on the diagnosis and early management of sepsis, severe sepsis, and septic shock were filtered using Medical Subject Heading (MeSH) terms and keywords. In the search, the terms "sepsis management," "septic shock management," "clinical sepsis treatment guidelines," "sepsis updated guidelines," "nano technology," "nano particles," and "antimicrobial resistance" were used. The review included prehospital and emergency department (ED) management of septic patients. In addition, we examined the most recent recommendations.

Search Strategy: Including MeSH terms and Keywords

The following terms and keywords were searched: “Septic Shock” OR “intravenous fluids” OR “antibiotics” OR “vasopressors” OR “corticosteroids” OR “lactate” OR “lactate clearance” AND Sepsis AND “Nanotechnology" OR “Nanoparticle.”

Inclusion and Exclusion Criteria

The papers found were filtered using the following inclusion criteria: papers written in English-language and articles published within the last 11 years were given preference. The papers were also ranked based on the type of research done, with updates and controversies of early sepsis management studies and advancements in nanotechnology receiving the most attention. In line with the study objectives, studies were also prioritized depending on the age of the patients, with adults receiving preference. Research addressing data from the past 11 or more years, and late therapy of sepsis and septic shock, nanotechnology importance not aimed at sepsis were among the studies that were excluded from the review.

Quality Appraisal

The selected articles were subjected to a quality assessment using a variety of available techniques. To identify any bias in the studies that could affect the overall quality of the clinical trials, the Cochrane Risk of Bias assessment method was used. The Newcastle Ottawa Scale was used to determine if the observational studies used in the study were biased. To assess the quality of systematic reviews, the Assessment of Multiple Systematic Reviews 2 tool was used. The quality of the results reported in the numerous studies used in the research was assessed using the Critical Appraisal Skills Randomized Controlled Trials Checklist.

Results

**Figure 1:**
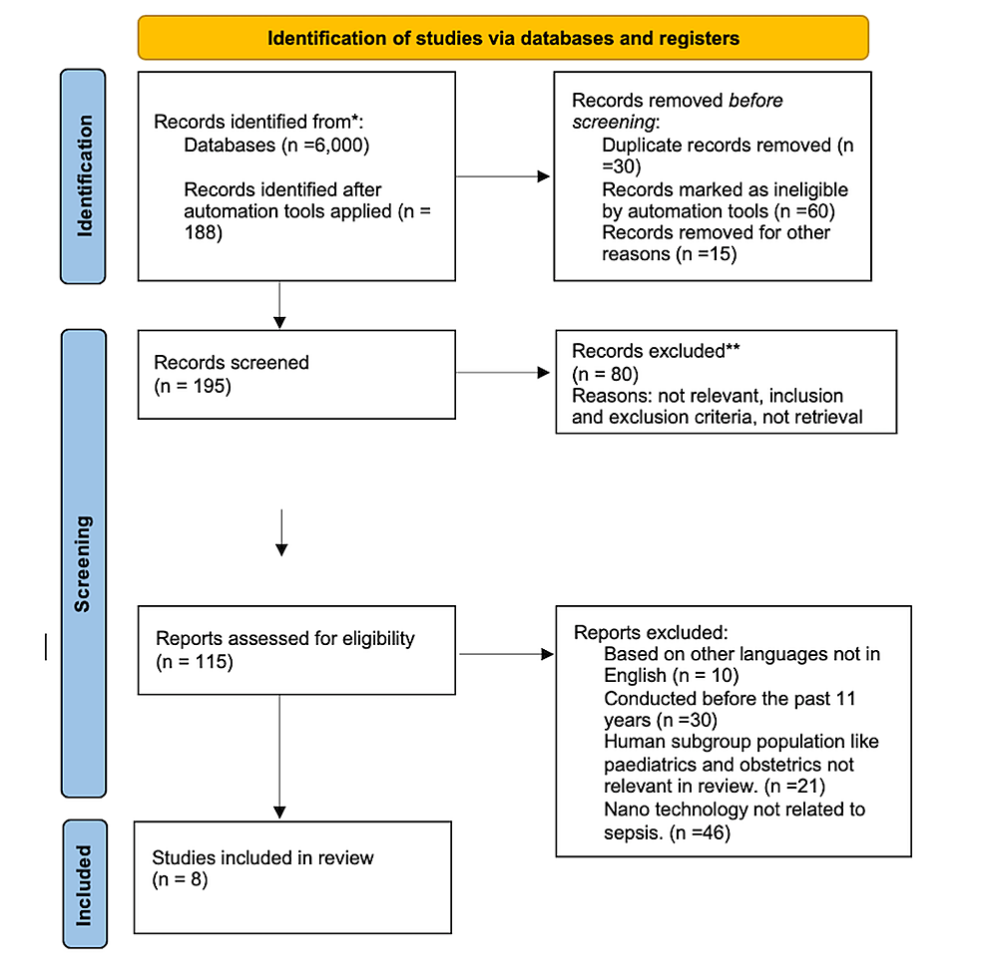
PRISMA flowchart PRISMA, Preferred Reporting Items for Systematic Reviews and Meta-Analyses

A total of 6,000 papers were found during the initial search for publications relevant to updates and controversies in early sepsis management, including nanosensors used in medication resistance for sepsis, which were then narrowed down to 195 articles relating to early sepsis and septic shock guidelines published between 2010 and 2021. The articles chosen for inclusion in the review were further screened to ensure that they were on adult people, excluding the obstetrics and pediatric populations, and that they were published in English. The application of nanotechnology in sepsis was also applied, and a total of eight articles were included. Figure 1 illustrates the PRISMA 2020 flow chart of article identification, displaying the many stages of the systematic review applied in the identification of studies.

Discussion

Systemic inflammatory response syndrome (SIRS), sepsis, and septic shock were initially defined in 1991. Sepsis was defined as SIRS due to infection (presumed or confirmed) and "septic shock" as septicemia with arterial hypotension despite adequate fluid resuscitation. The current definition acknowledges the severity and potential mortality of a disease caused by an invading virus and results in a process in which the body's defense response causes harm to itself [12].

An introduction to the pathophysiology of sepsis 

The pathogenesis of sepsis is thought to consist of a short-term hyperinflammatory phase followed by a longer-term immunosuppressive phase. The current death distribution demonstrates that there are peaks early on, but they are smaller in magnitude, and another peak after two to three months, which continues to increase over the next three years [13].

The etiology of sepsis also involves the early activation of both the innate and adaptive immune systems. During the early time, the overwhelming inflammatory reaction, also known as "cytokine storm," was responsible for the highest death rates resulting from fever, refractory shock, insufficient resuscitation, and cardiac or pulmonary failure. Meanwhile, persistent immunosuppression causes organ damage and/or failure, leading to late-period mortality [13].

Neutrophils are the most common and important type of innate cell produced in bone marrow and released into the bloodstream. They are essential for microbial elimination and sepsis survival, as well as key components of innate immunity. On their own, they participate in apoptosis or cell death. They die within 24 hours from an apoptosis that is both energy and caspase-dependent. Furthermore, due to delayed neutrophil apoptosis, the number of neutrophils increases significantly in the early stages of sepsis. Increased circulating neutrophils disrupt the immune system by releasing cytokines and reactive oxygen species (ROS) far from the infection site, resulting in multi-organ failure [13].

**Figure 2:**
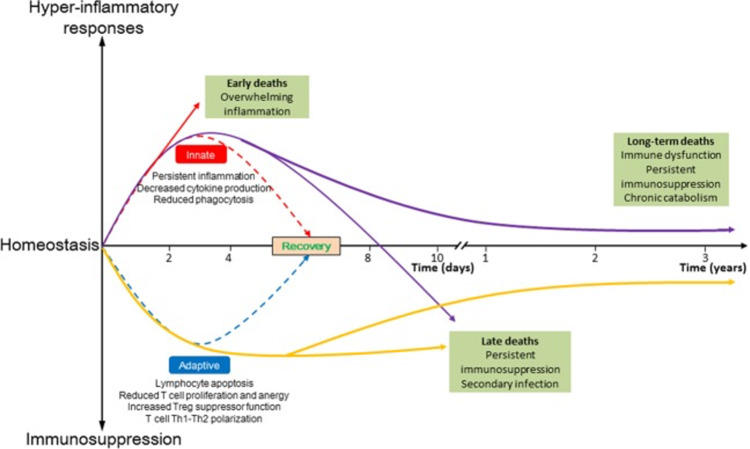
Activation of both innate and adaptive immune responses involved in the pathogenesis of sepsis Adapted from Cao et al. [13]. Copyright© Cao C, Yu M, and Chai Y (2019) Open Access This article is licensed under a Creative Commons Attribution 4.0 International License, which permits use, sharing, adaptation, distribution, and reproduction in any medium or format, as long as you give appropriate credit to the original author(s) and the source, provide a link to the Creative Commons license, and indicate if changes were made. The images or other third-party material in this article are included in the article’s Creative Commons license, unless indicated otherwise in a credit line to the material. If material is not included in the article’s Creative Commons license and your intended use is not permitted by statutory regulation or exceeds the permitted use, you will need to obtain permission directly from the copyright holder. To view a copy of this license, visit http://creativecommons.org/licenses/by/4.0/.

Figure 2 shows the aforementioned concept as well as highlights the early activation of both innate and adaptive immune responses involved in the pathogenesis of sepsis, as seen in the study by Cao et al. [13].

**Figure 3:**
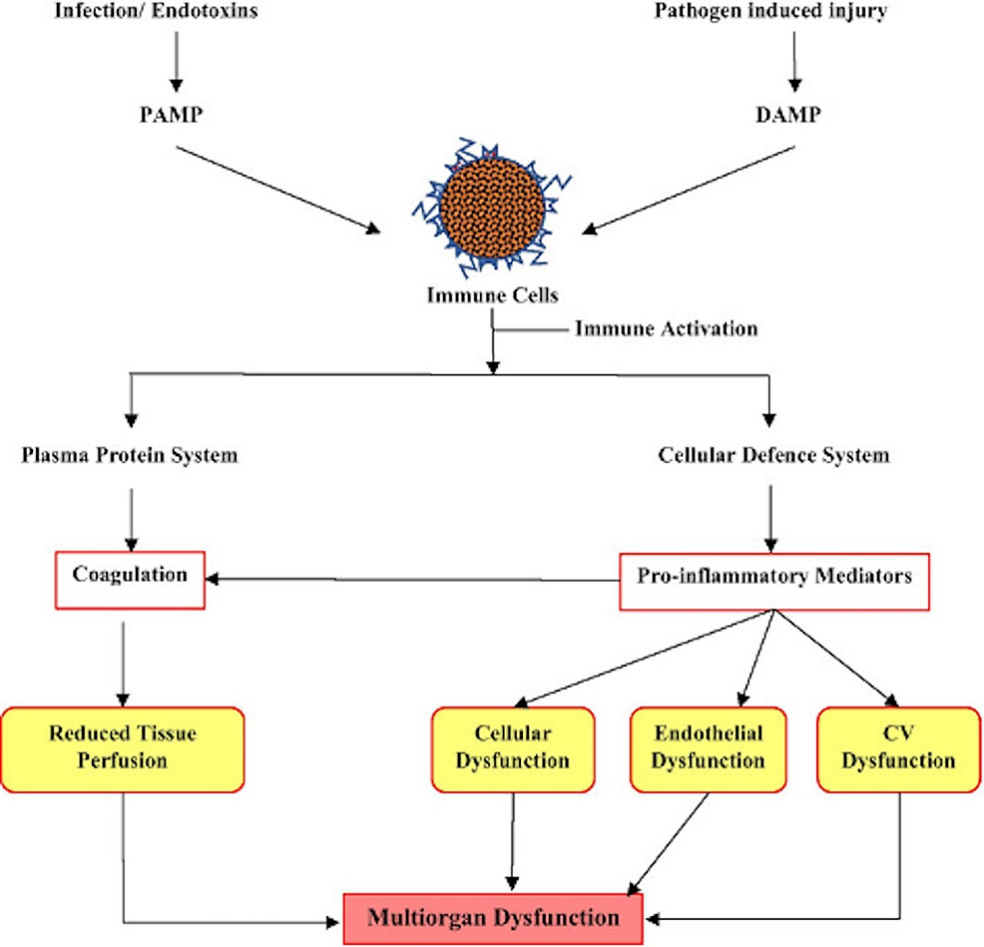
Pathophysiological processes that lead to sepsis Adapted from Pant et al. [2]. Copyright© Pant A, Mackraj I, and Govender T (2021) Open Access This article is licensed under a Creative Commons Attribution 4.0 International License, which permits use, sharing, adaptation, distribution, and reproduction in any medium or format, as long as you give appropriate credit to the original author(s) and the source, provide a link to the Creative Commons license, and indicate if changes were made. The images or other third-party material in this article are included in the article's Creative Commons license, unless indicated otherwise in a credit line to the material. If material is not included in the article's Creative Commons license and your intended use is not permitted by statutory regulation or exceeds the permitted use, you will need to obtain permission directly from the copyright holder. To view a copy of this license, visit http://creativecommons.org/licenses/by/4.0/. The Creative Commons Public Domain Dedication waiver (http://creativecommons.org/publicdomain/zero/1.0/) applies to the data made available in this article, unless otherwise stated in a credit line to the data. DAMP, damage-associated molecular pattern; PAMP, pathogen-associated molecular pattern

Figure 3 shows the numerous pathophysiological processes that lead to the activation of immune cells and the release of danger signal molecules (pathogen-associated molecular pattern [PAMP]/damage-associated molecular pattern [DAMP]), which then influence plasma protein and cellular responses. Organ dysfunction is caused by changes in blood flow and oxygen demand as a result of downstream processes, as seen in the study by Pant et al. [2].

Current method and diagnosis

Current national metrics and the Surviving Sepsis Guidelines propose the following for the early care of patients with sepsis and septic shock: During the first three hours, if possible, acquire two sets of blood cultures before starting antibiotics, give IV antibiotics within the first hour if possible, and offer an isotonic IV fluid challenge of 30 mL/kg to patients with hypotension or lactate greater than 4 mmol/L. For patients with a BMI of more than 30 kg/m2, ideal body weight is acceptable. Administer IV vasopressors to reach a mean arterial pressure (MAP) of at least 65 mm Hg during the first six hours, reassess intravascular volume status and tissue perfusion, and repeat lactate if initial levels are higher than 2 mmol/L [14].

If hypotension persists despite vigorous fluid therapy, one vasopressor infusion, such as norepinephrine or dopamine, is administered to keep MAP at 65 mmHg. Sazgar et al. conducted a clinical research with 138 septic shock patients. Norepinephrine was given to 70 patients, whereas dopamine was given to 68. At the start of the study, 30 minutes after the inotrope infusion, and 120 minutes after the inotrope infusion, the levels of end-tidal carbon dioxide (ETCO_2_), MAP, pulse rate (PR), and arterial blood gas (ABG) in two groups of patients were measured and compared. Norepinephrine-treated septic shock patients had a considerably greater ETCO_2_ rate and blood CO_2_ pressure than dopamine-treated patients. Despite the fact that key critical indicators such as MAP, O_2_ saturation, blood HCO_2_, blood PH, and mortality rate did not differ significantly between the two groups, patients receiving norepinephrine had a lower heart rate than those receiving dopamine. A low ETCO2 level has been associated with an increased risk of mortality in the hospital. Despite the fact that ETCO_2_ levels in both groups were the same at the start of the research and after 120 minutes, ETCO_2_ in the norepinephrine group increased considerably, with no influence on mortality. Patients' heart rates dropped throughout the therapy, although ETCO_2_ increased considerably in the norepinephrine group when compared to the dopamine group. This meant that tissue perfusion and shock fluid responsiveness were better in the norepinephrine group. According to other research, the death rate of patients receiving dopamine or norepinephrine for septic shock was not statistically significant; however, the dopamine group had more cardiac arrhythmias. As a result of the preceding evidence, both dopamine and norepinephrine are helpful in increasing microcirculation and tissue oxygen metabolism in the treatment of septic shock, with norepinephrine significantly exceeding dopamine in terms of clinical benefit [15].

In situations when norepinephrine is not accessible, epinephrine or dopamine can be utilized as a substitute. Individuals at risk of arrhythmias should be given additional attention while combining dopamine and epinephrine. Researchers propose giving vasopressin to individuals with septic shock who have low MAP levels instead of increasing their norepinephrine dosage. Vasopressin is not titrated to response like other vasopressors; instead, for the treatment of septic shock, it is generally administered at a fixed dosage of 0.03 units/minute. Vasopressin was used at a rate of 0.06 units per minute in clinical trials. Vasopressin has been associated with cardiac, digital, and splanchnic ischemia at larger doses. Although some evidence suggests that vasopressin is superior to norepinephrine in terms of clinical outcomes, the panel took into account the greater price and restricted availability of vasopressin and strongly recommended that norepinephrine be used initially. The guideline also considers the advantages and disadvantages of combining norepinephrine with vasopressin and makes a moderate recommendation for adding vasopressin rather than increasing norepinephrine dosage [14].

Dynamic evaluations have been demonstrated to be more accurate in predicting fluid response. Passive leg raising with cardiac output (CO) measurement, fluid challenges against stroke volume (SV), systolic pressure or pulse pressure, and increases in SV in response to intrathoracic pressure changes are all instances of dynamic measures. A systematic study and meta-analysis found that employing dynamic evaluation to guide fluid treatment was associated with lower mortality, ICU length of stay, and mechanical ventilation duration [14]. Douglas et al. investigated the effectiveness of dynamic measurements (SV change during passive leg raise) in guiding resuscitation and improving patient outcomes. The study also concluded that the use of the passive leg raise induced SV change to guide fluid and vasopressor resuscitation in the management of septic shock was deemed safe, with lower net fluid balance and a decreased risk of renal and respiratory failure. When compared to standard therapy, dynamic evaluations to guide fluid delivery may improve outcomes for patients with septic shock [16].

The administration of early antibiotic treatment as a critical variable in septic patients is confirmed by physicians. Rapid use of broad-spectrum antibiotics such as carbapenems or extended-spectrum lactamase inhibitor combination is strongly advised in the surviving sepsis recommendations [17].

The combination of glucocorticoids (GCs) and fludrocortisone decreased both short-term and long-term mortality in people with sepsis and septic shock. Although they increased the incidence of hyperglycemia, GCs reduced the time to shock resolution and the length of mechanical breathing. Although there was no good evidence to support the use of thiamine and ascorbic acid on a regular basis, they were associated with a few side effects [18].

Controversies 

Hemodynamic Controversies : Fluid management, Vasopressors, and Antibiotics in the Management of Sepsis

Fluid administration is fundamental in the management of hemodynamic instability, and optimizing its administration remains a challenge. Bolus administration of fluids may reduce arterial elastance, leading to vasodilatation and a hyperdynamic state, while excessive fluid administration is associated with organ dysfunction and death [12].

Normal saline solution has been used for decades, but concerns about side effects and acute kidney injury have sparked an interest in crystalloids, also known as chloride-restrictive solutions. Recent randomized clinical trials have been carried out to see which fluid administration is the most therapeutic in septic patients. Patients admitted to ICUs with sepsis who were given balanced solutions instead of normal saline had a lower 30-day mortality rate. To strengthen this claim, in a second trial with 1,641 patients admitted to medical ICUs with a sepsis, crystalloids were connected to decreased 30-day hospital fatality when compared to normal saline [12].

Fluid volume recommendations for various patient populations, such as congestive heart failure, end-stage renal disease (ESRD), and obesity, are widely debated. As per studies, fluid dosage based on modified body mass for obese individuals may be superior to fluid dosing based on actual or ideal body weight. It is also possible that people with congestive heart failure and ESRD will be treated with lower fluid intake; however, no differences in mortality have been found. In hypotensive patients or those with lactate levels greater than 4 mmol/L, the first liquid portions should generally adhere to 30 mL/kg quality metrics, with the assessment in the evaluation of specific patient's fluid demands taking priority over treatment guidelines [19].

In Roberts et al.’s study, the researchers wanted to see if there was a link between vasopressor dosing intensity and a 30-day in-hospital mortality during the first 6-24 hours after the onset of septic shock, if the effect of vasopressor dosing intensity varies by fluid resuscitation volume, and if the effect of vasopressor dosing intensity varies by dosing titration pattern. According to the results, increasing the potency of vasopressors during the first 24 hours after septic shock was linked to an increase in mortality. This relationship changed depending on how much early fluid was given and when the vasopressor was titrated [20].

Endotracheal intubation of septic patients with etomidate has been controversial for a long time. Although etomidate is known to cause adrenal suppression, the extent to which this is clinically significant is unknown [21]. A recent study of septic patients intubated with etomidate compared to ketamine discovered that the etomidate group experienced significantly more hypotension 6 to 12 hours after intubation than the ketamine group. A prior study discovered a link between etomidate use with an increased mortality and adrenal insufficiency in sepsis. The study, however, had significant methodological flaws. Notably, patients who had previously received etomidate were excluded from the current ADRENAL study, highlighting the investigators' concerns that etomidate could cause clinically significant adrenal suppression [22,23]. Most experts believe that etomidate should be regarded as a second-line induction drug in light of alternatives such as ketamine in the early management of sepsis and septic shock [23].

Antibiotic therapy is essential in the treatment of sepsis, and improper administration during the first 24 hours is associated with an eight-fold increase in in-hospital mortality. Previous studies have shown that insufficient empirical antibiotic therapy was associated with a 74% increase in inflammatory response progression. Other difficulties linked with antibiotic therapy include hemodynamic changes, subtherapeutic doses, and regional differences in resistance patterns [24,25].

Despite the use of supportive care and prompt dosing, antibiotics are frequently ineffective and have little influence on decreasing sepsis mortality. Sepsis-induced immune paralysis predisposes critically sick patients to secondary infections, especially infections caused by multidrug-resistant (MDR) bacteria. As a result, in addition to antibiotic medication and routine supportive therapies, these patients require particular tactics aimed at restoring immune response function. As a result, these adjuvant medicines can benefit the immune system by avoiding immune paralysis or reducing inflammatory reactions [26].

To strengthen the above argument, Zhang et al. focused on adult individuals with acute sepsis or septic shock. It was concluded that time to adequate antibiotic treatment was found to be an independent driver of post-infection ICU and hospital lengths of stay in patients with sepsis. To enhance results and shorten the length of illness, clinicians should establish local measures aimed at rapid delivery of effective antibiotic medication [17].

GC-resistance in sepsis (GCR) is a well-known symptom of sepsis that may contribute to GC failure to help sepsis patients. GCR refers to the GC's inability to control the transcription of GR-responsive genes despite apparent acceptable plasma cortisol concentrations. In septic shock, there was evidence of a link between the degree of GC unresponsiveness and illness severity and death [27].

Other perspectives

Currently, there are no unique treatments for sepsis, although there are various prospective therapies and new research areas to look into. The recent Rapid Treatment of Carnitine in Septic Shock (RACE) experiment examined the hypothesis that L-carnitine administration could minimize cumulative organ failure in septic shock patients. Another trial aiming to treat sepsis with the help of vitamin C and other fluids found that patients who received this innovative medication had a lower fatality rate [28].

Patients who require ventilators or oxygen treatment are clearly in need of specialized care, while other patients at risk of advancing from sepsis to septic shock may need to be admitted to the ICU. As a result, it is vital to prepare for anticipated ICU needs ahead of time, as some research shows that patients admitted to a general medicine floor and later moved to ICUs have poor outcomes [29,30].

Intensive care for patients with terminal illnesses may not be in accordance with their individual and family goals for care. When resuscitation efforts are unsuccessful or go against the patient's wishes, lenient directives should be considered. However, allowing natural death directions does not exclude cardiopulmonary resuscitation. A comparison of the expected outcomes of several treatment options might aid patients and decision-makers in deciding which course is best [30].

Advancement in sepsis: can nanotechnology play a pivotal role in sepsis diagnosis and management?

Serum analysis and molecular methods have traditionally been used to diagnose sepsis. Conventional diagnostic procedures are expensive and time-consuming, and lack acceptable sensitivity and selectivity, necessitating the development of alternative sepsis diagnosis techniques. Nanosensors, on the other hand, offer the much-appreciated potential for accurate sepsis diagnosis [3].

Nanobiotechnology advancements have created a new concept for biosensor systems with enhanced capabilities. A perspective on nanotechnology applications and its proactive role is discussed next [3].

What is Nanotechnology?

Nanosensors based on electrochemical, immunological, or magnetic principles allow for a sensitive, selective, and a quick detection of sepsis biomarkers such as procalcitonin and C-reactive protein [2,3].

Surface functionalization with antimicrobial peptides improves effectiveness even further by targeting bacteria or particular microenvironments. Several methodologies in nanoformulations have been studied that have shown the capacity to administer antibiotics and anti-inflammatory drugs at the same time. The importance of nanoformulations of additional adjuvant medicines, such as antioxidants, antitoxins, and extracorporeal blood purification in the therapy of sepsis, is also emphasized. As evidenced by Pant et al., inclusion of prospective future biomedical uses, nanodiagnostics, and nanotherapeutics in sepsis hold enormous promise and open up new avenues for sepsis care [2].

Nanomaterials are small enough to fit between biomolecules and cells. In theory, NPs with design optimization can modulate cell state and function. As a result, researchers have been intrigued by the use of nanomaterials in sepsis treatment over the past two decades. Advances in nanotechnology have also enabled the creation of complex nanostructures with distinct physical characteristics and surface chemistry that offer several potential advantages over conventional medicinal agents [31].

Thus, research has been set out to provide a comprehensive understanding of the application of nanomaterials to the pathophysiology of sepsis, as well as nanotherapies for reducing inflammation and reactive oxygen and nitrogen species (RONS) in sepsis [31].

Over recent decades, sepsis treatment has evolved. The intensivists are in charge of deciding which management alternatives to use. The importance of early detection and treatment of this illness has improved results in management. As a consequence of enhanced public understanding, donations and money invested in sepsis research and technology have surged. The concern is whether bundles are still necessary given how much our understanding of sepsis has progressed. Aside from innovative medicine and complementary therapies, further research is needed into establishing earlier source control and conservative versus liberal oxygen therapy and the usefulness of these factors in improving sepsis care [8].

Figure 4 from Chen et al. shows that microorganisms enter the circulation and concentrate in large numbers in lesion sites, causing significant inflammation and the production of RONS via the innate immune system's activation. This will result in blood vessel leakage, severe organ failure, and perhaps death. As a result, in the therapy of sepsis, limiting immune system activation and lowering inflammation and RONS are crucial. Nanomaterials offer a breakthrough in the treatment of sepsis and are classified as follows: inhibition of pattern recognition receptor (PRR) signaling pathways, nanomaterials for RONS elimination, nanomaterials for inflammatory elimination, and multifunctional nanomedicine [31].

**Figure 4:**
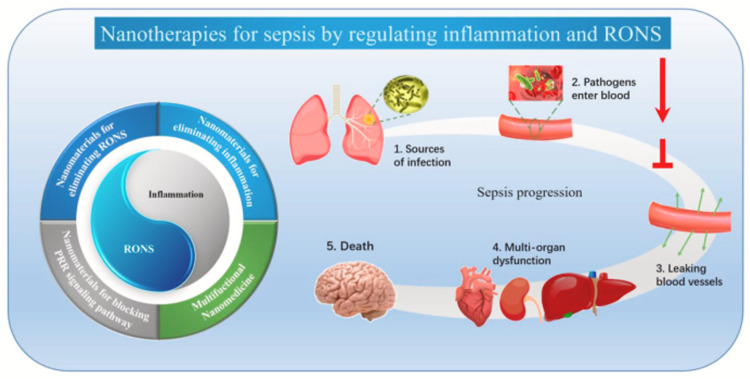
Nanomaterials affecting different pathway Adapted from Chen et al [31]. Copyright© Chen L, Huang Q, Zhao T, Sui L, Wang S, Xiao Z, Nan Y, and Ai K (2021) This is an open access article under the CC BY-NC-ND license (http://creativecommons.org/licenses/by-nc-nd/4.0/). RONS, reactive oxygen and nitrogen species

Excessive RONS in sepsis considerably surpass the scavenging activity of antioxidant enzymes in vivo causing oxidative stress in the body, which creates additional RONS. As a result, it is vital to rely on exogenous antioxidants to maintain the body's redox equilibrium. Traditional antioxidants have a number of drawbacks such as side effects, quick elimination, and limited stability and bioavailability, which restrict their clinical applicability. Recent research has discovered that nanomaterials have significant promise in the treatment of sepsis by directly eliminating RONS or providing traditional antioxidants, which not only overcome the drawbacks of traditional antioxidants but also increase clinical effectiveness [32-34].

**Figure 5:**
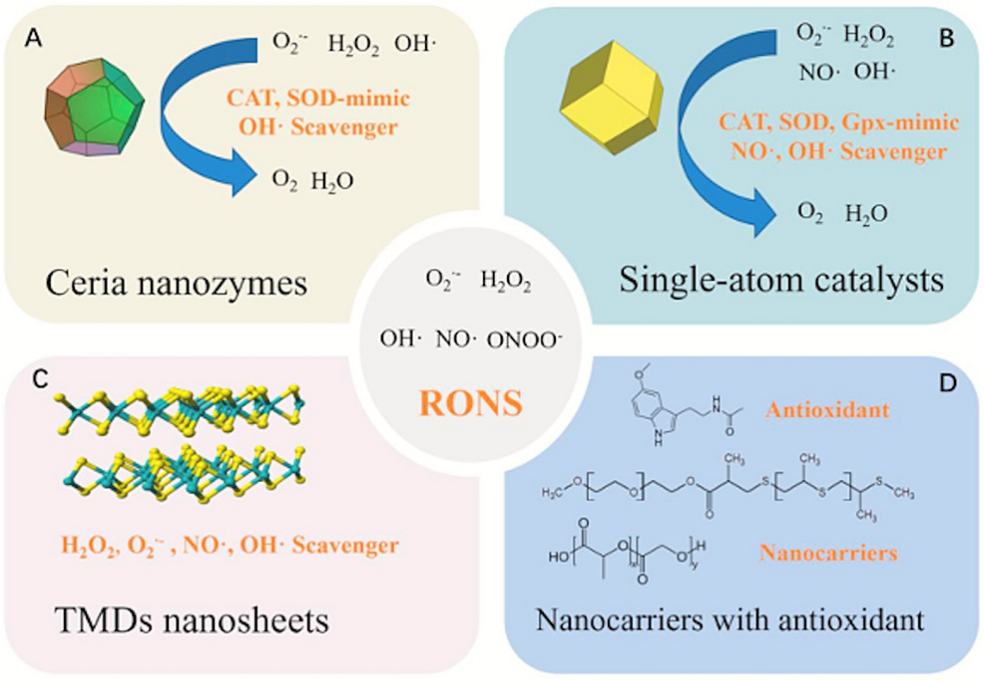
Nanomaterials cleave reactive oxygen species, a mediator of sepsis Adapted from Chen et al. [31]. Copyright© Chen L, Huang Q, Zhao T, Sui L, Wang S, Xiao Z, Nan Y, and Ai K (2021) This is an open access article under the CC BY-NC-ND license (http://creativecommons.org/licenses/by-nc-nd/4.0/). TMD, transition-metal dichalcogenide

Figure 5 is from Chen et al.’s study illustrating how nanomaterials such as Ceria nanozymes and single-atom catalysts can remove excessive RONS in vivo by simulating numerous antioxidant enzymes. Two-dimensional transition-metal dichalcogenide (2D-TMD) nanosheets may efficiently scavenge different RONS to cure sepsis, and nanomaterials can function as carriers delivering antioxidants to the site of inflammation [31].

PRRs are proteins that recognize compounds found in infections known as PAMPs or molecules produced by damaged cells known as DAMPs. PAMPs and DAMPs can start a vicious cycle between the inflammatory response and RONS by identifying PRRs on immune cells or non-immune cell membranes. When PAMPs or DAMPs bind to PRRs, the vicious cycle begins. As a result, using specific nanomaterials to target signaling pathways for sepsis therapy is a successful strategy [35].

In the later stages of sepsis, a prolonged or severe low inflammatory state can result in immune effector failure, which can lead to immunosuppression. As a result, sepsis therapy necessitates inflammation control. Inflammation is commonly treated with anti-inflammatory medications, both steroidal and nonsteroidal. However, they are ineffective in the treatment of sepsis. Anti-TNF or anti-IL-1 monoclonal antibodies, as well as other medications, have limited anti-sepsis therapeutic benefits [36-37].

Nanomaterials can be carefully designed to alter the expression of pro-inflammatory and anti-inflammatory molecules and prioritized to reach target tissues from the site of administration, overcoming the problems of off-target organ side effects and systemic toxicity. Many different types of nanomaterials have been developed to control inflammation in sepsis, including nanocarrier devices for anti-inflammatory medications, anti-inflammatory nanomaterials, and nanomaterials that actively target inflammatory cells [31].

Fenton et al. focused on resolvins, protectins, and maresins, and their corresponding derivatives. These substances induce neutrophil death by allowing nonphlogistic macrophages to remove debris and dead cells and begin tissue healing. These specific mediators have been shown to be effective in a variety of chronic inflammatory illness models. The research emphasized how biomaterial resolvin D2 has been proven to eliminate local and systemic bacterial load and reduce excessive inflammation, thus controlling the immune response without immunosuppression and thereby resolving sepsis [38].

In vivo, polymer NPs quickly produce enhanced aggregation and provide a danger of toxicity. Cerium oxide, gold NPs (auNPs), iron oxide, 2D-TMD (WS2, MoS2), and silica are examples of inorganic nanomaterials used to treat sepsis. In particular, iron oxide and molybdenum disulfide are made up of components found in the human body [38-39].

Biocompatible and biodegradable in vivo polymer NPs are commonly used. By regulating the composition, stability, responsiveness, and surface charge of nanocarrier-based polymers, drug loading and release kinetics may be precisely controlled. In vivo, polymer NPs can quickly generate enhanced aggregation and pose a toxicity concern [40].

Research by Hou et al. shows how antimicrobial resistance has added to the difficulty of treating sepsis, necessitating the development of new therapeutic techniques such as nanotechnology. Researchers demonstrated how adoptive transfer of macrophages expressing antimicrobial peptides linking to cathepsin B in the lysosomes (MACs) can be used to treat MDR bacteria-induced sepsis in immunocompromised mice. Antimicrobial peptide and cathepsin B (AMP-CatB) mRNA is transduced into vitamin C lipid NPs to produce MACs. The vitamin C lipid NPs enable the particular accumulation of AMP-CatB in macrophage lysosomes, where high antimicrobial actions are carried out. The study concluded that the process of MAC transfer eliminates MDR bacteria such as Staphylococcus aureus and Escherichia coli, allowing immunocompromised septic mice to recover completely [41].

In sepsis, the presence of increased neutrophil extracellular traps (NETs) has been investigated. NETs are physiologically important because they can immobilize and destroy a wide spectrum of infections. NETs cause organ damage by interfering with tissue function, thrombosis, and the autoimmune system. In the extracellular space of neutrophils, NETs are frequently linked with citrullinated histones (CitH3). In animal models of sepsis, strategies that target NET development or NET components have been shown to be effective. Protein arginine deiminases (PAD) inhibitors have been found to protect animals from endotoxic shock or septic shock by disrupting NET formation [42].

Lee et al.’s work focused on target-triggered aggregation or dissociation of superparamagnetic iron oxide nanoparticles (SPIONs). These particles have been used to detect a variety of biomarkers via magnetic relaxation switching (MRSw). However, there has never been a documented MRSw-based biosensor for ROS [43]. Research shows how PEGylated bilirubin (PEG-BR)-coated SPIONs were made via simple sonication and ligand exchange. They were loaded with a near-infrared fluorescent dye for fluorescence-based ROS detection. The NPs were able to directly detect the amounts of ROS in whole blood samples in a sepsis-mimetic clinical environment. The project was completed. These findings imply that PEG-BR@SPIONs might be utilized to identify sepsis-related diseases with ROS overproduction as a novel form of dual-mode (MRSw-based and fluorescence-based) biosensor for ROS detection [43].

Lipopolysaccharide (LPS) is a component of the outer membrane of gram-negative bacteria that may activate surface receptors upon interaction. These interactions cause a significant systemic inflammatory response by triggering the progressive production of a range of proinflammatory cytokines such as IL-8, IL-1, and IL-6. Mishra used a lipid emulsion containing ciprofloxacin that was created to treat intra-abdominal infections, particularly sepsis. The proportions of chitosan and sodium deoxycholate affected loading efficiency. The findings suggest that chitosan can change how LPS interacts with macrophages and that formulations could help to decrease sepsis-related inflammation [44].

Research by Weiss et al. discussed how nanomaterials have been used to fine-tune immune responses and how their properties might be used to prevent cytokine release. Nanosystems can improve the specificity and efficiency of immunosuppressive drug delivery to target immune cells, resulting in lower medication doses, less drug distribution to non-target tissues and organs, and fewer adverse effects. Furthermore, particular nanotools may be created to elude the immune system and improve the solubility of poorly soluble immunosuppressant medicines; the ability to carefully tune their surface charge opens up encapsulation methods and allows for a large drug load to be accommodated. All of these processes might occur together, increasing the effectiveness of immunosuppressive drugs [45].

Given their regulatory qualities and ability for surface functionalization, the use of NPs for diagnostic and therapeutic purposes has a lot of promise. Magnetic NPs (MNPs), AuNPs, fluorescent NPs (silica and quantum dots), and lipid-based NPs, as well as technologies such as lab on a chip, point of care (POC), and biosensor, have all been researched to allow the identification of sepsis-related microbial infections [10].

Nanotechnology can thus play an important role in more current times, especially in the COVID-19. COVID-19 possesses pathological features that are similar to those of classical sepsis, and severe COVID-19 has been labeled as viral sepsis. Progress in sepsis research is critical for improving these patients' clinical outcomes. Recent developments in our knowledge of sepsis' pathophysiology have led us to believe that key causes are unregulated inflammatory response and oxidative stress [31]. One key characteristic of COVID-19's is the induction of a cytokine storm in the body, also known as cytokine release syndrome (CRS), which is caused by an overactive immune response causing significant deterioration of health. This inflammatory storm is one of the main causes of acute respiratory distress syndrome (ARDS), which is typically linked to sepsis-related multi-organ failure and is one of the leading causes of mortality in critically ill patients [45].

However, aside from the therapeutic benefit, the toxicity of nanotherapies in clinical translation is a major issue. Nanomaterial toxicity is complicated by a number of factors, including their composition, size, surface charge, and distribution in vivo. Also, NETs linked to histones can cause organ damage by interfering with tissue function, thrombosis, and the autoimmune system [38,42,43].

Table 1 lists researches discussed throughout the article relevant to nanotechnology and its implication in sepsis diagnosis and management.

**Table 1:** Tabulated series of nano technology in the application of sepsis diagnosis and management AMP-CatB, antimicrobial peptide and cathepsin B; CatB, cathepsin B; CH, chitosan; LPS, lipopolysaccharide; MACs, macrophages with antimicrobial peptides connected to cathepsin B in the lysosomesMDR, multidrug-resistant; mRNA, messenger RNA; MRSw, magnetic relaxation switching; NETs, neutrophil extracellular traps; PAD, protein arginine deiminases; QD, quantum dot; ROS, reactive oxygen species; SDC, sodium deoxycholate; SPIONs, superparamagnetic iron oxide nanoparticles; VCLNP, vitamin C lipid nanoparticle; YOP, year of publication

Author	YOP	Aim of study	Nanomaterial used	Role of nanomaterial	Outcome and conclusion	
Lim et al. [10]	2021	Nanomaterials importance in sepsis diagnosis and management	gold NPs, fluorescent NPs (silica and QDs), and lipid-based NPs	Fluorescent signal amplification, resistant, targeting DNA bacterial organism	Promising vital tool in diagnosing and treating sepsis	
Fenton et al. [38]	2018	Importance of biomaterials for drug delivery	Resolvin D2	Apoptosis of neutrophils	Resolvin D2 has been shown to minimize excessive inflammation and eradicate local and systemic bacterial burden, hence modulating the immune response without immunosuppression and curing sepsis	
Hou et al. [41]	2020	Vitamin lipid NPs enable adoptive macrophage transfer for the treatment of MDR bacterial sepsis	VCLNP	The AMP-CatB mRNA is given to the macrophage by way of a VCLNP. CatB cleaves mRNA in the lysosomes after it is translated in the cytoplasm. The ingested MDR germs are eliminated by the pre-stored AMp-IB367 when phagosomes containing MDR bacteria merge with the lysosomes	It was theorized that adoptive transfer of MACs might help the immunocompromised sepsis host increase innate immunity, avoid bacterial immune evasion, and eradicate MDR pathogens. Immunocompromised septic mice recover completely.	
Deng et al. [42]	2020	Citrullinated histone H3 as a therapeutic target for endotoxic shock in mice	Citrullinated histone H3	NETs	NET development or NET components have been shown to be effective. PAD inhibitors have been found to protect animals from endotoxic shock or septic shock by disrupting NET formation.	
Lee et al. [43]	2020	Targeted aggregation or dissociation of SPIONs causes MRSw	SPIONs	Magnetic switching relaxation	(MRSw- and fluorescence-based) biosensors for ROS detection might be utilized to identify a variety of disorders linked to excessive ROS generation-like sepsis.	
Mishra [44]	2011	Function of target LPS in sepsis treatment through polymeric capped nano-structured formulation	CH and SDC	LPS interaction with macrophages	CH can change how LPS interacts with macrophages, and preparations incorporating it have the potential to decrease sepsis-related inflammation.	
Weiss et al. [45]	2020	The role of nanotechnology-enabled approaches against the COVID-19 pandemic	Different nanomaterials	Cytokine inhibition	May reduce sepsis-induced multi-organ failure	
Zhu et al. [46]	2014		Oligonucleotide-conjugated SPION	Bacterial infection and drug resistance diagnosis for sepsis	It is possible that this will cause a delay in the timely detection and treatment of infection-related emergencies such as bacterial sepsis	

Limitations

The research used in this analysis, which is heavily reliant on examining publications, may be limited, resulting in a lack of understanding about the subject. Furthermore, a large number of articles included in this review focused on controversies and updates on sepsis and septic shock diagnosis and early care, leaving secondary variables, which are extremely important, unaddressed. Additional restriction is that only articles published in the last 18 years are allowed, with more focus on sepsis guidelines and application of nanotechnology in the last 11 years. As most of the investigations were based on previous research, the foundation of knowledge might well be constrained, as there may be older studies with data that might considerably contribute to the study. Additionally, clinical development of antimicrobial nanotechnologies remains difficult. Nanotechnology has several limitations despite the fact that it is still being explored. Changes in shape and size, for example, can result in a variety of physical and chemical interactions; a material that is non-toxic at a certain nano may become poisonous at another size, and vice versa. NP hazards and dangers include increased formation of ROS, DNA damage, genotoxic effects, organ and tissue damage in humans and animals, and environmental toxicity. Thus, most studies remain in the trial phase and remain difficult to be tested on humans due to their toxicities. The fast introduction of novel biomaterials and nanomaterials will necessitate a more thorough assessment of their biocompatibility.

## Conclusions

Sepsis is a fatal disease that has been recognized worldwide. It is a reaction that occurs when the host's immune system is damaged as a result of the body's aggressive response to an infection. The pathogenesis of sepsis is linked to inflammation and an excess of RONS, which activate the PAMP-PRR and DAMP-PRR signaling pathways. Rapid treatment of sepsis necessitates urge identification, antibiotic management, careful hemodynamic aid, and treatment of the implicit infection.

Nanotechnology, a subset of nanomedicine, may aid in the development of a rapid, sensitive, and precise sepsis diagnosis. MNPs have all been discussed as potential tools for detecting microbial infections associated with sepsis. This study discusses recent advances in nanotechnology-based sepsis detection and management technologies. Additionally, developing more clinically relevant animal models, identifying microbial pathogenesis mechanisms and new biomarkers, understanding the microenvironment of bacterial infection sites, and lowering regulatory barriers can all contribute to the successful translation of antimicrobial nanotechnologies. Traditional conservative management and diagnosis are challenging, but nanotechnology may be able to overcome them. While nanotechnology is driving a number of advances in the antibacterial sector, the clinical development of antimicrobial nanotechnologies remains challenging. The rapid introduction of novel biomaterials and nanomaterials that have been clinically proven to be ineffective will necessitate a more thorough assessment of their biocompatibility and long-term safety.
